# The antigenic property of the H5N1 avian influenza viruses isolated in central China

**DOI:** 10.1186/1743-422X-9-148

**Published:** 2012-08-06

**Authors:** Wei Zou, Jianjiang Ke, Jiping Zhu, Hongbo Zhou, Meilin Jin

**Affiliations:** 1 State Key Laboratory of Agriculture Microbiology, Huazhong Agriculture University, Wuhan, P. R, 430070, China; 2 Laboratory of Animal Virology, College of Veterinary Medicine, Huazhong Agriculture University, Wuhan, P. R., 430070, China

**Keywords:** Avian influenza virus, Antigenic epitope, Antigenic drift

## Abstract

**Background:**

Three influenza pandemics outbroke in the last century accompanied the viral antigen shift and drift, resulting in the change of antigenic property and the low cross protective ability of the existed antibody to the newly emerged pandemic virus, and eventually the death of millions of people. The antigenic characterizations of the viruses isolated in central China in 2004 and 2006–2007 were investigated in the present study.

**Results:**

Hemagglutinin inhibition assay and neutralization assay displayed differential antigenic characteristics of the viruses isolated in central China in two periods (2004 and 2006–2007). HA genes of the viruses mainly located in two branches in phylogeny analysis. 53 mutations of the deduced amino acids of the HA genes were divided into 4 patterns. Mutations in pattern 2 and 3 showed the main difference between viruses isolated in 2004 and 2006–2007. Meanwhile, most amino acids in pattern 2 and 3 located in the globular head of the HA protein, and some of the mutations evenly distributed at the epitope sites.

**Conclusions:**

The study demonstrated that a major antigenic drift had occurred in the viruses isolated in central China. And monitoring the antigenic property should be the priority in preventing the potential pandemic of H5N1 avian influenza virus.

## Background

Three influenza pandemics in 20th century (1918 H1N1 Spanish, 1957 H2N2 Asian and 1968 H3N2 Hong Kong) and the first influenza pandemic in 21st century (H1N1/2009 Mexico) were due to the direct interspecies transmission or exchange of gene segments between avian, swine and human influenza viruses [1]. The newly emerged pandemic strains were antigenically divergent from seasonal influenza viruses circulating at that time. Vaccines efficient for the seasonal flu could not elicit any cross-reactivity in humans. Tens of thousands of people died in each pandemic due to the lack of effective cross-protection of existed antibody.

HA protein is the primary target of neutralizing antibodies and continuously accumulates mutations to escape recognition of the immune system. Alteration of the antigenic epitopes of HA protein results in immune evasion and more rapid spread of influenza virus. The antigenic epitopes of H3 subtype influenza virus were well characterized and mapped to the three dimensional structure of the HA protein [2,3]. The epitopes of H5 avian influenza virus (AIV) were also identified through sequencing HA gene of the escape mutants selected by specific monoclonal antibodies (Mabs) [4-6]. Nearly all amino acids in epitopes located in the surface of the HA protein.

In our previous study, an H5N1 highly pathogenic AIV (HPAIV), A/duck/Hubei/hangmei01/2006 (hm/06), had been isolated from brains of dead laying ducks with severe central nervous system (CNS) dysfunction [7]. Subsequently, several HPAIV H5N1 viruses isolated from ducks and pigeons also showed neurovirulence in field ducks and pigeons. In view of the increasing virulence as well as mortality to the natural host, waterfowls, we try to elucidate whether the changed biological properties are related to the antigenicity of these H5N1 viruses isolated after 2005. Our previous study had identified the antigenicity of the viruses isolated in 2004 [8]. The present study compared the antigenic features of the viruses isolated in 2004 and 2006–2007 in central China. Hemagglutination inhibition (HI) and neutralization assay (NT) activity, the phylogenetic tree and deduced amino acids of HA gene as well as the location of mutated sites in the HA protein crystal model were performed to reveal the molecular mechanism of the antigenic properties of the viruses isolated respectively from the two periods in central China.

## Results

### 2.1 The HI activity of the Mabs to the 10 viruses

**Figure 1:**
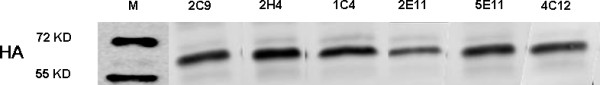
**Reactivity of the Mabs to the HA protein of virus dw/04.** Due to the different Mabs were used to perform the western blot, the six reactions were performed separately and the image was resembled together. Same HA protein loading and exposure time were used in the western blot assay.

Before detecting the HI activity of the Mabs to the 10 viruses, western blot assays were used to identify the activity of the selected Mabs. The results displayed that all six Mabs could recognize the HA protein of virus dw/04 (figure 1). Then the Mabs were tested for their abilities to inhibit hemagglutination of chicken erythrocytes to the selected 10 viruses (table 1). Mab 2 C9 showed moderate HI activity to all the 10 viruses. But the other five Mabs displayed obviously weaker HI activity to the viruses isolated in 2006–2007 than those isolated in 2004. Mab 5E12 displayed relatively higher HI activity to all the 10 selected viruses, however, the difference in HI activity could be observed in the two periods (table 1).

**Table 1:** Comparison of antigen city of the 10 viruses in NT and HI assays using 6 HA protein Mabs

**H5N1 strains**	**2 C9**		**2 H4**		**1 C4**		**5E12**		**2E11**		**4 C12**	
	**NT**	**HI**	**NT**	**HI**	**NT**	**HI**	**NT**	**HI**	**NT**	**HI**	**NT**	**HI**
zfe/04	30720	128	9486	1024	30720	16384	30720	16384	378	128	11943	64
dw/04	35481	32	12800	512	30720	16384	30720	8192	672	64	6400	64
xfy/04	102400	32	32359	512	102400	16384	102400	16384	724	64	27542	32
xfj/04	74131	256	436	64	102400	16384	102400	16384	977	64	31623	64
ewhc/04	102400	<16	45709	256	102400	16384	102400	16384	12800	32	4467	32
hm/06	30720	64	192	256	156	256	1503	512	<60	<16	<60	<16
d16/06	30720	<16	168	128	2799	1024	13095	1024	<60	<16	405	<16
xn/07	28840	32	<200	<16	<200	64	2754	256	<200	<16	<200	<16
zg/07	27542	<16	<200	<16	1738	1024	16596	4096	<200	<16	457	<16
xg/07	20283	256	<60	<16	<60	32	600	512	<60	<16	<60	<16

### 2.2 The NT activity of the Mabs to the 10 viruses

The Mabs displayed different neutralizing efficiency to the viruses isolated from the two periods (table 1). Mab 2 C9 displayed extremely high neutralization activity to all the 10 viruses, which was inconsistent with the moderate HI activity. This incoordination might indicate that Mab 2 C9 did not bind to the receptor binding regions of the HA protein. The NT activity of the four Mabs (2 H4, 1 C4, 5E12 and 4 C12) is much higher to the 5 viruses isolated in 2004 than those isolated in 2006–2007. However, the Mab 2E11 displayed very low NT activity to 10 AIV H5N1 strains except the virus ewhc/04.

### 2.3 The phylogenetic analysis of the HA gene

**Figure 2:**
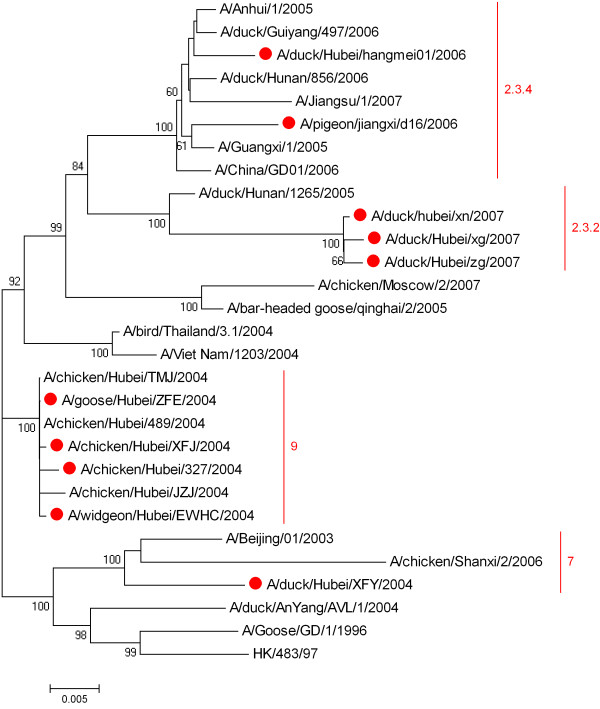
**Phylogenetic analysis of HA gene of the 10 viruses.** The phylogenetic tree was build with the program MEGA 4.1. Clades of H5N1 virus were classified according to nomenclature recommended by WHO evolution working group. The viruses used in present study were labeled by red spot.

Phylogenetic analysis was performed to elucidate the evolution situation of the viruses isolated in the two periods. The viruses mainly formed two different branches in the phylogenetic tree. And the viruses isolated in 2004 were closely related to the viruses isolated in Vietnam. The HA clade except xfy/04 was 9, while the HA clade of xfy/04 was 7 (figure 2). The viruses isolated in 2006–2007 belonged to Fujian-like sublineage and the HA clade of hm/06 and d16/06 was 2.3.4 and the HA clade of zg/07, xg/07 and xn/07 was 2.3.2 (figure 2).

### 2.4 Analysis of the mutated amino acids in HA protein of the viruses

To further investigate the molecular foundation of the antigenic difference of the 10 viruses, amino acid mutations of HA protein were analyzed. A total of 53 amino acid substitutions appeared in the HA protein of the selected 10 viruses (table 2). According to the distribution of the mutated amino acids, the 53 substitutions could be divided into four different patterns. In pattern 1, the substitutions mainly happened in virus xfy/04, and the other 9 viruses were basically homologous; in pattern 2, substitutions happened in viruses xg/07, xn/07 and zg/07, which were isolated in 2007; in pattern 3, substitutions happened in all the 5 viruses isolated in 2006–2007; and in pattern 4, substitutions happened sporadically in all the viruses (table 2).

**Table 2:** Mutated amino acid sites of the deduced HA protein of the 10 viruses

**sites**	**zfe**	**dw**	**xfy**	**xfj**	**ewhc**	**hm**	**d16**	**xn**	**zg**	**xg**^**a)**^	**note**^**b)**^
21	E	E	***V***^***c)***^	E	E	E	E	E	E	E	Pattern 1
114	I	I	***V***	I	I	I	I	I	I	I	
119	K	K	***R***	K	K	K	K	K	K	K	
188	T	T	***I***	T	T	T	T	T	T	T	
189	R	R	***K***	R	R	R	K	R	R	R	
282	M	M	***V***	M	M	*I*	*I*	M	M	M	
320	S	S	***A***	S	S	S	S	S	S	S	
325	R	R	***G***	R	R	R	R	R	R	R	
418	F	F	***S***	F	F	F	F	F	F	F	
450	D	D	***E***	D	D	D	D	D	D	D	
483	R	R	***K***	R	R	R	R	R	R	R	
499	K	K	***N***	K	K	K	K	K	K	K	
511	I	I	***M***	I	I	I	M	I	I	I	
513	T	T	***I***	T	T	T	T	T	T	T	
2	Q	Q	Q	Q	Q	Q	Q	***H***	***H***	***H***	Pattern 2
40	K	K	K	K	K	K	K	***R***	***R***	***R***	
45	D	D	D	D	D	D	D	***N***	***N***	***N***	
53	R	R	K	R	R	R	R	***K***	***K***	***K***	
120	S	S	S	S	S	S	S	***D***	***D***	***D***	
129	S	S	S	S	S	S	S	***L***	***L***	***L***	
154	N	N	N	N	N	N	N	***D***	***D***	***D***	
156	T	T	T	T	T	T	T	***A***	***A***	***A***	
162	R	R	R	R	R	R	R	***K***	***K***	***K***	
184	A	A	A	A	A	A	A	***E***	***E***	***E***	
200	V	V	V	V	V	V	V	***I***	***I***	***I***	
226	M	M	M	M	T	M	M	***I***	***I***	***I***	
263	A	A	A	A	A	A	A	***T***	***T***	***T***	
277	K	K	K	K	K	K	K	***R***	***R***	***R***	
528	A	A	A	A	A	A	A	***V***	***V***	***V***	
533	V	V	V	V	V	V	V	***M***	***M***	***M***	
84	S	S	S	S	S	***N***	***N***	***N***	***N***	***N***	Pattern 3
94	D	D	D	D	D	***N***	***N***	***N***	***N***	***N***	
124	N	N	N	N	N	***D***	***D***	***D***	***G***	***D***	
138	L	L	L	L	L	***Q***	***Q***	***Q***	***Q***	***Q***	
155	S	S	S	S	S	***N***	***N***	***N***	***N***	***N***	
227	E	E	E	E	E	***D***	***D***	***D***	***D***	***D***	
269	L	L	L	L	L	***V***	***V***	***V***	***V***	***V***	
310	R	R	R	R	R	***K***	***K***	***K***	***K***	***K***	
35	K	K	K	K	K	K	K	R	K	K	Pattern 4
139	G	*R*	G	G	G	G	G	G	G	G	
140	K	K	R	K	K	*T*	*T*	N	N	N	
141	S	S	*P*	S	S	*P*	*P*	S	S	S	
151	I	I	I	I	I	I	I	I	I	T	
161	K	K	K	K	K	*R*	K	K	K	K	
174	V	V	V	V	V	*I*	*I*	V	V	V	
181	P	P	P	P	P	*S*	*S*	P	P	P	
209	L	*F*	L	L	L	L	L	L	L	L	
240	N	*D*	N	N	N	N	N	N	N	N	
313	L	L	L	L	L	*P*	L	L	L	L	
322	Q	Q	Q	Q	Q	*L*	*L*	Q	Q	Q	
354	Y	Y	Y	Y	Y	Y	*F*	Y	Y	Y	
366	A	A	A	T	A	A	*A*	A	A	A	
467	C	C	C	C	C	*R*	C	C	C	C	

Note:

a): abbreviations of the 10 viruses: zfe/04, dw/04, xfy/04, xfj/04, ewhc/04, hm/06, d16/06, xn/07, zg/07 and xg/07.

b): the four patterns mutated amino acids according to mutated situation showed in the text.

c): residues in bold plus italic plus underline were mainly pattern 1 mutation happened in virus xfy/04; residues in bold plus italic were mainly pattern 2 and 3 mutations happened in viruses isolated in 2006–2007; residues in italic were sporadic mutations in the 10 viruses.

### 2.5 Distributions of the mutated amino acids in the HA crystal structure

**Figure 3:**
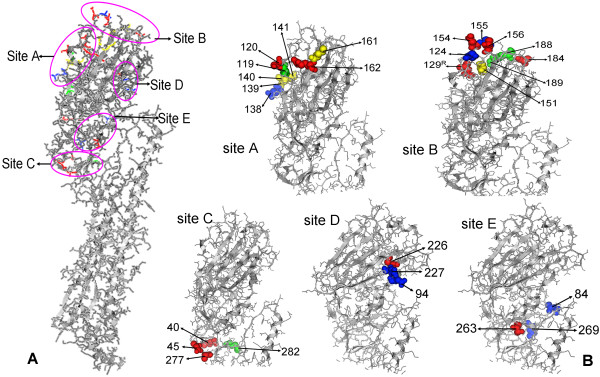
**Mutated sites in the crystal structure of HA protein.** The crystal model is HA protein of A/Vietnam/1203/2005, built by the program PyMOL program (V1.4). **A** is the intact model of HA protein displayed the mutated amino acids in the five antigenic epitope sites. **B** is the detail profile of the five antigenic epitope sites A, B, C, D and E. The mutated amino acid sites in the 10 avian influenza viruses HA proteins located in the five epitopes were labeled. Amino acids labeled with green were pattern 1 mutation; amino acids labeled with red were pattern 2 mutation; amino acids labeled with blue were pattern 3 mutation; amino acids labeled with orange were pattern 4 mutation (table 2).

The precise sites of the mutated amino acids were mapped to the crystal structure of AIV HA molecule, 3GBM. The model displayed that 36 of the 53 mutations located in the globular head of HA1 proteins. And it could find that amino acid sites 119, 120, 138, 139, 140, 141,161and 162 were located at site A epitope of the HA protein; amino acid sites 124, 129, 151, 154, 155, 156, 184, 188 and 189 were located at site B epitope; amino acid sites 40, 45, 277 and 282 at site C epitope; and amino acids 94, 226, 227 at site D epitope; amino acids 84, 263, 269 at site E epitope (figure 3).

## Discussion

The H5N1 AIVs continuously outbreak in East Asia countries after 2005 and form a new phylogenetic clade, gaining some new characterizations, such as neurovirulence and the ability to infect poultry and migratory birds [7,9]. Meanwhile, most of the subsequent viruses isolated from the infected human in China prove to be clade 2.3 [10]. H5N1 HPAIVs cause a constant increase of human infection cases and nearly 60% of the infected people die ( http://www.who.int/influenza/human_animal_interface/H5N1_cumulative_table_archives/en/index.html). Meanwhile, H5N1 HPAIVs are raging all over the world in poultry as well as migrated birds and putting significant threat to human health and poultry breading.

Further analysis reveal that the HA gene of these viruses belong to Clade 2.3. The properties of the sequences, virulence and even the transmission property of the viruses in this clade are investigated profoundly in many reports [7,11-15]. Wu et.al show that viruses isolated from 2002 to 2007 have formed four distinct antigenic groups, and imply that a major antigenic drift might happen in the viruses isolated after 2005 (clade 2.3) [16]. But the antigenicity of the viruses in this clade is not fully studied. Investigation about the antigenicity of H5N1 AIVs isolated in different time and geographical areas after 2005 is beneficial to clarifying the changed biological properties of the emerged viruses.

In the present study, combining the HI and the NT activity of the Mabs to the 10 selected viruses isolated in central China, we conclude that a significant antigenic drift have occurred in the viruses isolated in the two periods. To reveal the molecular mechanism of the antigenic change, phylogenic analysis is performed and displays that HA genes of the viruses isolated in two periods obviously locate in different branches. The deduced amino acids demonstrate that the 53 substitutions in the HA protein of the 10 viruses are divided into four patterns. The pattern 1 mutations are only observed in virus xfy/04, the same sites in other 9 viruses are identical. Our previous study had demonstrated that the virus xfy/04 showed low pathogenicity in mice, chickens and pigeons, while the other 9 viruses showed high pathogenicity in animal experiments or in field [7,17]. Therefore, pattern 1 mutations might be related to the low pathogenicity of virus xfy/04.

The amino acids in pattern 3 mutations are different in viruses isolated in 2004 and 2006–2007, indicating that this pattern mutation might happen from 2005 to 2006. However, the mutations in pattern 2 are only observed in the viruses isolated in 2007, indicating that this pattern mutation might happen from 2006 to 2007. This information reveals that the antigenicity of H5N1 AIV is constantly changing in the process of spread. On the other hand, viruses isolated in 2006 (hm/06 and d16/06) belong to clade 2.3.4, while viruses isolated in 2007 (xn/07, zg/07 and xg/07) belong to clade 2.3.2. Meanwhile, viruses in clade 2.3.2 have gradually replaced the viruses in clade 2.3.4 in these years and become the most prevalent viruses in China. This might suggest that viruses undergoing antigenicity alteration have a great advantage in prevalence and spread.

## Conclusions

The present study reveals that the antigenic epitopes of the HA protein have changed in the prevalent AIVs in central China. The mutations in pattern 2 and 3 might be responsible for the differences of HI and NT activity and the distinct antigenic features of the viruses isolated in the two periods. Particularly, most mutations in pattern 2 and 3 locate in the globular head and distribute at the five epitope sites, and even represent the trend of antigenic drift in 2004–2007. This phenomenon might demonstrate that those sites actually expose to the selected pressure and easily cause antigenic drift. Generally, the H5N1 AIVs are undergoing further antigenic drift and persistent monitoring the epidemiology and antigenic evolution of H5N1 AIV is imperative.

## Materials and Methods

### 4.1 Virus propagation, purification and titration

The H5N1 AIV used in this study included: A/Chicken/Hubei/327/2004 (dw/04), A/Goose/Hubei/ZFE/2004 (zfe/04), A/Chicken/Hubei/XFJ/2004 (xfj/04), A/Duck/Hubei/XFY/2004 (xfy/04), A/widgeon/Hubei/EWHC/2004(ewhc/04), A/duck/Hubei/hangmei01/2006 (hm/06), A/pigeon/jiangxi/d16/2006 (d16/06), A/duck/Hubei/xn/2007 (xn/07), A/duck/Hubei/zg/2007 (zg/07), and A/duck/Hubei/xg/2007 (xg/07). All H5N1 strains were propagated in 9-day-old SPF embryonated eggs. Allantoic fluids were harvested and stored at −80°C. TCID_50_ titrations of all ten AIV isolates were performed according to Reed and Muench in MDCK cultures [18].

### 4.2 Production of monoclonal antibodies against dw/04

The panel of Mabs (2 C9, 2 H4, 1 C4, 5E12, 2E11 and 4 C12) against HA protein of dw/04 was stored at −80°C in our lab. The protocol of the Mab production had been previously described [8]. The antigenic characterization and neutralization assay of the viruses isolated in 2004 had been characterized in previous study [8].

### 4.3 Western blot assays

The Mabs activities were analyzed by western blot assay with HA protein of dw/04. 5 μg purified HA protein was dissolved in the loading buffer and applied onto the gels. Following the gel electrophoresis, the separated proteins were transferred to nitrocellulose membrane (Boster, China) using Trans-Biorad SD (Bio-Rad, USA). Each membrane was washed once with TBS-T buffer for 10 mins and blocked with 5% skimmed milk at 4°C overnight and then incubated with the Mab at 37°C for 1 h. Finally, after triple washes, the membrane was incubated with horseradish peroxidase-conjugated anti-mouse IgG (Boster, China) at 37°C for 1 h and visualized with ECL western-blotting detection system (Amersham, USA).

### 4.4 HI assay

HI assay of the Mabs were performed as described previously, using chicken erythrocytes for agglutination and four hemagglutination units of the selected 10 viruses [19].

### 4.5 Virus neutralization assays

Two-fold serial dilutions of the Mabs were mixed with the virus suspensions (100 TCID_50_ per well) and incubated together for 1 h at 37°C. Then 100μL mixture was added onto washed Madin-Darby canine kidney (MDCK) cell in a flat bottom 96-well cell culture plate (Corning Incorporated Costar, USA). Cytopathogenic effect (CPE) was observed after a 3-day incubation at 37°C in the presence of 5% CO_2_. Neutralizing titers were expressed as the highest dilution of the virus which could result in 50% neutralization of the CPE.

### 4.6 Phylogenetic analysis of the HA genes of the viruses

Total RNA was extracted from the allantoic fluid using Rneasy mini kit (Qiagen, California, USA), and reverse transcribed into cDNA. All the HA genes of selected viruses were amplified by the specific HA oligonucleotide primers. RT-PCR and sequencing of the HA genes of the viruses were repeated to confirm that the sequence data were identical. The other HA gene sequences for phylogenetic analysis were obtained from the NCBI Database. Sequences of HA gene were edited and aligned by ClustalX (Version1.81) software and phylogenetic tree was produced by MEGA 4.0.2 software.

### 4.7 Analysis of the mutated amino acid sites in the HA crystal model

The deduced amino acids were aligned to further analyzed the mutated sites of the HA protein of the 10 viruses (DNAStar, Madison, WI). The crystal structure data of HA protein of the virus A/Vietnam/1203/2004 was download from PDB data bank ( http://www.rcsb.org/pdb/home/home.do) and the PDB ID was 3GBM. All the mutated amino acids in HA proteins of the 10 viruses were mapped to the crystal model of protein 3GBM with the program PyMOL program (V1.4). The 5 antigenic sites of H5N1 AIV referenced previous reports or blasted the HA genes to the H3 subtype AIV whose epitopes had mapped to the crystal model of HA protein [2-5,8,16,20-22].

## Abbreviations

AIV: avian influenza virus; CPE: cytopathogenic effect; HI: hemagglutination inhibition; HPAIV: highly pathogenic avian influenza virus; Mab: monoclonal antibody; MDCK: Madin-Darby canine kidney; NT: neutralization assay; TCID50: 50% tissue culture infection dose.

## Competing interests

The author(s) declare that they have no competing interests.

## Authors' contributions

W Z and M J designed the experiments; W Z, J K and J Z performed the experiments; J K, J Z, H Z sequenced the HA genes of the viruses, performed the phylogenetic analysis and contributed the reagents and materials; W Z and M J wrote the manuscript. All authors read and approved the final manuscript.
